# Reactive and Senescent Astroglial Phenotypes as Hallmarks of Brain Pathologies

**DOI:** 10.3390/ijms23094995

**Published:** 2022-04-30

**Authors:** Andrijana Lazic, Vanda Balint, Danijela Stanisavljevic Ninkovic, Mina Peric, Milena Stevanovic

**Affiliations:** 1 Laboratory for Human Molecular Genetics, Institute of Molecular Genetics and Genetic Engineering, University of Belgrade, Vojvode Stepe 444a, 11042 Belgrade, Serbia; vanda@imgge.bg.ac.rs (V.B.); danijelastanisavljevic@imgge.bg.ac.rs (D.S.N.); minaperic@imgge.bg.ac.rs (M.P.); milenastevanovic@imgge.bg.ac.rs (M.S.); 2 Faculty of Biology, University of Belgrade, Studentski trg 16, 11000 Belgrade, Serbia; 3 Serbian Academy of Sciences and Arts, Kneza Mihaila 35, 11001 Belgrade, Serbia

**Keywords:** astrocytes, senescence, reactive astrogliosis, neurodegenerative diseases, SASP, astrocyte-targeted therapy, in vitro models

## Abstract

Astrocytes, as the most abundant glial cells in the central nervous system, are tightly integrated into neural networks and participate in numerous aspects of brain physiology and pathology. They are the main homeostatic cells in the central nervous system, and the loss of astrocyte physiological functions and/or gain of pro-inflammatory functions, due to their reactivation or cellular senescence, can have profound impacts on the surrounding microenvironment with pathological outcomes. Although the importance of astrocytes is generally recognized, and both senescence and reactive astrogliosis have been extensively reviewed independently, there are only a few comparative overviews of these complex processes. In this review, we summarize the latest data regarding astrocyte reactivation and senescence, and outline similarities and differences between these phenotypes from morphological, functional, and molecular points of view. A special focus has been given to neurodegenerative diseases, where these phenotypic alternations of astrocytes are significantly implicated. We also summarize current perspectives regarding new advances in model systems based on astrocytes as well as data pointing to these glial cells as potential therapeutic targets.

## 1. Introduction

For decades, traditional neurocentric view has dominated the research of brain functions. Accordingly, pathology and diseases of the human brain have been generally assigned to malfunction or the loss of neurons. In contrast, the roles of astrocytes have been largely underestimated and, for many years, astrocytes were considered to provide only passive support to neurons. However, knowledge about astrocytes, and their participation in numerous aspects of the central nervous system’s (CNS) physiology and pathology, has continuously expanded over the last two decades [1]. At present, it is known that this the most abundant glial cell type in the brain exhibits numerous roles necessary for the coordinated functioning of CNS [2]. Astrocytes play an active role in neuronal transmission and synaptic plasticity [3] and control ionic homeostasis, neurotransmitter levels, metabolic supply, and blood flow [4]. Through numerous processes specific to astrocyte morphology, they form close interactions with neurons and other glial cells [5]. Astrocytes are an indispensable part of synaptic transmission, as proposed by tripartite as well as tetra- and pentapartite synapse models [6,7]. As suggested by Semyanov and Verkhratsky [8], astrocytes, microglia, oligodendrocytes, blood vessels, extracellular space, and the extracellular matrix form an active milleu where neuronal activity not only propagates among pre- and postsynaptic neurons, but also provides signals to the other cellular and noncellular compartments which affect the nervous tissue [8]. Astrocytes respond to neuronal activity with an elevation of their internal Ca^2+^ concentration, which triggers the release of chemical transmitters from glia themselves and, in turn, causes the feedback regulation of neuronal activity and synaptic strength [9]. In synapses, astrocytes take up neurotransmitters such as glutamate, gamma-aminobutyric acid (GABA), adenosine and norepinephrine, and maintain their extracellular concentrations and recycling [10]. Glutamate acts as major excitatory neurotransmitter in the CNS [10], and its untimely clearance from synaptic space can cause glutamate excitotoxicity, neuronal hyperexcitation, and damage [11]. Glutamate clearance is managed through Na^+^- and K^+^-dependent [12] excitatory amino acid transporters 1 (EAAT1) and 2 (EAAT2), expressed in astrocytes, which are human homologues for rat glutamate transporters, the glutamate aspartate transporter (GLAST), and glutamate transporter 1 (GLT-1), respectively. The uptake and recycling of glutamate in astrocytes are dependent on extracellular potassium concentrations [13]. In periods of neuronal activity, the extracellular concentration of potassium increases. Astrocytes express the inwardly rectifying potassium channel 4.1 (Kir4.1) responsible for potassium uptake, and through the process of spatial buffering maintain extracellular potassium homeostasis [14,15]. Astrocytes redistribute potassium from regions of high potassium concentration towards regions with low potassium and blood vessels [14].

Astrocytes are important components of the blood–brain barrier (BBB). The BBB is formed by the capillary endothelial cells and is surrounded by the basal lamina and astrocytic perivascular endfeet. It has several specialized characteristics, including the high expression of Kir4.1 and water channel aquaporin 4 (AQP4), which are involved in ion and volume regulation [16]. The barrier also plays important roles in the homeostatic regulation of the brain microenvironment, and in communication between the nervous system and the periphery [17], thus, it is necessary for the healthy function of the CNS [18]. AQP4 is enriched in astrocyte endfeet at the synapses and at the glia limitans, where it mediates water exchange across the BBB and controls cell volume, extracellular space volume, astrocyte migration [19], and the maintenance of brain fluid homeostasis [20]. The perivascular enrichment of AQP4 has also been shown to be required for the proper functioning of the glymphatic system [19,21]. The glymphatic waste clearance system, presented by perivascular channels formed by astroglial cells, eliminates soluble proteins and metabolites from the CNS and helps in the distribution of non-waste compounds such as glucose, lipids, amino acids, and neurotransmitters [22]. The dysfunction of the glymphatic system and waste removal are implicated in age-related diseases [22]. The cerebrospinal fluid inflow of larger tracers and contrast clearance in glymphatic system have been shown to be reduced in the human brain tissue of elderly people and in aged wild-type mice [23,24,25,26]. The decrease in the glymphatic flow in old mice is, in part, mediated by the redistribution of the AQP4 water channels away from the vascular wall and astrocytic endfeet towards the cell soma [24], and possibly by the atrophy of meningeal lymphatic vessels [27]. Reduced glymphatic clearance might be predictive of increased risk of protein aggregation, given the combination of locally stagnant fluid flow together with the elevated extracellular concentration of the aberrant proteins [28].

Astrocytes provide the cellular link to the neurons in the neurovascular unit, interact with the smooth muscles and endothelial cells on the micro vessels, and are involved in blood supply and the regulation of local blood flow [18]. Astrocytes contact blood vessels where they take up glucose and supply synapses and different regions of neurons with glucose and lactate [2,29], thus, they represent important providers of energy metabolites in the CNS. These glial cells are also the main sites of glycogen storage, which is utilized during periods of high neuronal activity and hypoglycemia [30,31]. The importance of astrocytes in the energy metabolism of CNS is emphasized by the astrocyte–neuron lactate shuttle hypothesis (ANLS) [32], which explains the role of lactate as an important energy source for brain function and defines the strong metabolic association between astrocytes and neurons. Under intensified periods of neuronal activity, lactate serves as an alternative energy substrate [33]. Lactate produced by astrocytes and secreted via monocarboxylate transporters 1 (MCT1) and 4 (MCT4) is taken by neurons via the monocarboxylate transporter 2 (MCT2) [33]. On the other hand, the hypothesis proposed by Bak et al. argues that neurons use lactate as an ‘opportunistic’ substrate whenever it is present, rather than during neurotransmission activity [34,35]. Changes in energy metabolism have been implicated in CNS disease and aging [33,36].

Given the importance of astrocytes in mechanical, metabolic, and trophic support to the neurons, the loss of astrocyte homeostatic functions (or the gain of pro-inflammatory functions), as a result of their reactivation or cellular senescence, has profound implications in various CNS pathologies, including neurodegenerative diseases (NDs). Apart from these detrimental functions, reactivated astrocytes also exert many functions beneficial for neural tissue recovery, including: the secretion of neurotrophic factors with neuroprotective functions; protection from oxidative stress by clearing reactive oxygen species (ROS) [37]; the limitation of inflammation spread; and the restoration of ion homeostasis and the reconstruction of the BBB [38,39]. Although the importance of astrocytes is generally recognized, their complex physiology and their protective and adverse effects on the surrounding microenvironment are still incompletely understood. In this review, we summarize the latest data regarding astrocyte reactivation and senescence. Since cumulating evidence points to these alterations in astrocyte phenotypes as underlining mechanisms of different NDs, the resulting brain pathologies are also included in this review. We emphasize the complexity of both astrocyte reactivation and senescence and outline similarities and differences between them. We also summarize current perspectives regarding new advances in model systems based on astrocytes, as well as data indicating these glial cells as potential therapeutic targets.

## 2. Reactive Astrogliosis

Reactive astrogliosis is a defensive reaction of astrocytes, which can be caused by virtually all pathological conditions in the brain including trauma, brain aging, ischemia, bacterial or virus infections, and different NDs [40,41]. For a long time, reactive astrogliosis has been viewed as a stereotypic process that exerts mostly detrimental effects on neural tissue preservation and the repair upon CNS injury. Today, it is known that reactive astrogliosis is a complex, gradated process which, depending on the severity of the injury, includes variable changes in gene expression, cellular proliferation and hypertrophy, and in severe cases leads to scar formation [2,41]. For decades, the glial scar was thought to be a primary inhibitor of CNS recovery, preventing the adaptive neural plasticity underlying the recovery of function. However, this concept has been continually challenged and presently it is increasingly accepted that the glial scar plays a dual role in CNS recovery: both protective and inhibitory. In fact, glial scars are predominately formed by reactivated astrocytes that can gain many beneficial functions in order to support neural tissue recovery, restore brain homeostasis, and limit tissue damage, but they can also gain negative functions that have detrimental effects on neuron survival and axon regeneration [38,40,42,43,44,45].

Although astrocyte reactivity is generally associated with microglial reactivity and leukocyte recruitment, this review will focus on astrocytes and will not specifically address the role of reactive microglia and immune cell infiltration in coordinated and complex processes of neuroinflammation.

### 2.1. Morphological and Molecular Features of Reactive Astrocytes

**Figure 1:**
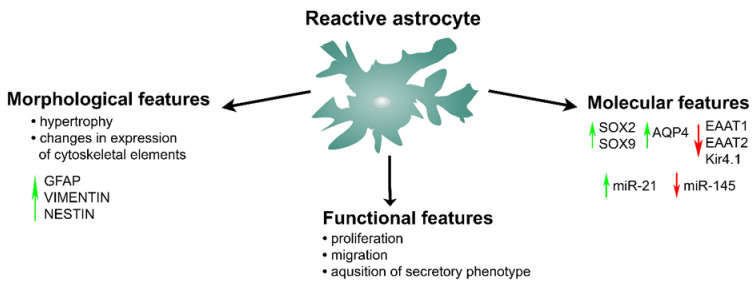
Major morphological, molecular, and functional features of reactivated astrocytes. Reactive astrocytes undergo morphological changes in response to injury, including variable levels of hypertrophy (of cell body and processes) depending on the severity of the injury, as well as the increased expression of intermediate filaments, i.e., glial fibrillary acidic protein (GFAP), VIMENTIN, and NESTIN. In more severe cases of CNS injury, reactive astrocytes proliferate and migrate toward the site of injury where they form a glial scar. They acquire secretory phenotypes with either beneficial or detrimental effects on CNS recovery. Reactive astrocytes go through significant molecular expression changes. They up-regulate neural stem cell-related factors SOX2 and SOX9. They down-regulate astrocytic glutamate transporters, excitatory amino acid transporter 1 and 2 (EAAT1, EAAT2), as well as inwardly rectifying potassium channel 4.1 (Kir4.1) and up-regulate the aquaporin 4 (AQP4) water channel. miR-21 is up-regulated, while miR-145 is down-regulated in reactive astrocytes.

During reactive astrogliosis, astrocytes undergo a wide set of morphological, functional, and genetic changes that can influence the functional outcome of all CNS disorders (Figure 1). The most prominent morphological characteristics of a reactive astrocyte are the hypertrophy of the cell body and the increased expression of glial fibrillary acidic protein (GFAP) [40,43]. Although the underlying mechanisms of these morphological changes need further clarification, independent studies have indicated the important regulatory role of the signaling pathway Janus kinase (JAK)/signal transducer and the activator of transcription protein 3 (STAT3) in these phenotypic alternations [40,46]. In particular, studies using knockout mice or the conditional deletion of the *Stat3* gene showed that the absence of this gene led to disrupted up-regulation of GFAP expression, the failure of astrocyte hypertrophy, and attenuated astroglial scar formation, which all caused poor outcomes after CNS injury [46,47]. Besides GFAP, both VIMENTIN and NESTIN, the intermediary filaments characteristic of the earlier stages of astrocyte development [48,49], are up-regulated in reactive astrocytes [41,50,51]. Interestingly, the reactive phenotype of astrocytes differs depending on: the type of injury, the region of the CNS affected by the injury, and the localization of reactive astrocytes in respect to the injury [52,53]. Specifically, GeneChip analysis performed on mice by Zamanian LJ et al. showed that the gene expression changes in reactive astrocytes vary tremendously depending on the type of injury. In particular, the up-regulation of at least 50% of genes differs depending on whether the reactive phenotype was induced by neuroinflammation or ischemic stroke. For example, GFAP and VIMENTIN are up-regulated in astrocytes by both neuroinflammation and ischemia, but NESTIN-positive cells are seen only during neuroinflammation [52].

Reactive astrocytes also undergo profound functional changes, including the acquisition of secretory phenotypes with either beneficial or detrimental effects (Figure 1). These cells perform their beneficial functions by secreting a wide set of neuroprotective molecules. For example, they exert their anti-oxidative functions by secreting molecules such as glutathione [54]. They also secrete a wide array of neurotrophic factors such as glia-derived neurotrophic factor (GDNF), ciliary neurotrophic factor (CTNF), brain-derived neurotrophic factor (BDNF), and nerve growth factor (NGF), aiding the growth and survival of neurons [55]. Interestingly, astrocytes are the main endogenous producers of anti-inflammatory cytokine, transforming growth factor beta (TGFβ), which has neuroprotective functions in several CNS pathologies [56]. They also have an important role in restoring the brain ion homeostasis and the BBB in different pathological conditions [38,39]. Besides positive effects, reactive astrocytes can gain secretory functions detrimental for CNS recovery. In reaction to a diverse set of effector molecules, astrocytes secrete a wide array of different molecules such as cytokines, including interleukins: IL1β, IL6, IL11, and IL15; tumor necrosis factor alpha (TNFα); leukemia inhibitory factor (LIF); chemokines: CXCL12, CXCL1, CXCL9, CXCL10, CCL2, CCL7, CCL8 and CCL5; matrix metalloproteinase 3 (MMP3), and others [53]. These molecules can promote CNS inflammation by recruiting and instructing the cells of the immune and inflammatory response [53,57,58]. The acquisition of secretory phenotype is a complex coordinated process governed by different mechanisms including the p38 mitogen-activated protein kinase (MAPK)/nuclear factor-κB (NF-κB) pathway [59,60]. It has been shown that the inhibition of p38 protein leads to the attenuation of activity of the NF-κB transcription factor, which subsequently suppresses the transcription of its target genes and diminishes the inflammatory response, leading to better outcomes during CNS repair [60]. NF-κB has been proposed to have dual roles in different pathological conditions of the CNS [57]. This transcription factor induces the release of pro-inflammatory factors from astrocytes that promote neuroinflammation and, therefore, create an environment that is not favorable for neural repair. On the other hand, NF-κB stimulates the production of neurotrophinsby astrocytes, which favor neuron survival [57].

Characteristic functional changes of reactive astrocytes include increased proliferation and migration towards the site of injury, where they form a glial scar (Figure 1) [61]. These changes are usually seen in more severe forms of CNS injury, and are also recognized features of astrocyte precursors during CNS development [38,50,61]. Although reactivated astrocytes and glial precursors share some phenotypic properties, it is not completely understood if they are governed by the same molecular mechanisms. However, there are indications that gene expression profiles in reactive astrocytes recapture those specific for astrocyte precursors and neonatal astrocytes present during brain development [62,63]. Thus, it has been shown that the SOX2 transcription factor, which has the main role in the maintenance of the high proliferative capacity of astrocytes during development, is also up-regulated in reactive astrocytes [64]. Interestingly, the re-expression of *Sox*2 in reactive astrocytes is correlated with their reacquired proliferation ability [64]. Additionally, another member of the SOX family—the SOX9 transcription factor, which is crucial for the initiation of gliogenesis [65]—has been shown to be up-regulated in reactivated astrocytes upon injury [66]. However, the downstream molecular events in these cells triggered by this transcription factor are still not fully understood (Figure 1).

There is growing evidence indicating that miRNAs—small, regulatory RNAs that negatively regulate gene expression at the post-transcriptional level—have important regulatory roles in astrocyte reactivation [67,68,69]. In particular, it has been shown that miR-21, which is up-regulated during reactive astrogliosis, induces profound positive changes in astrocyte responses to injury [70]. On the other hand, miR-145, which is expressed in astrocytes in physiological conditions, is down-regulated during reactive astrogliosis [71]. Different studies show that, apart from miR-21 and miR-145, many other miRNAs have an altered expression during different CNS injuries, where they exert important roles in the regulation of reactive astrogliosis [67,68,69].

The essential alterations induced by astrocyte reactivation include the down-regulation of the glutamate transporters and the Kir4.1 channel that work together to facilitate glutamate uptake [13]. These changes, and the subsequent excitotoxicity, appear as events that favor the development of amyotrophic lateral sclerosis and other NDs, as discussed later in this review. On the other hand, the expression of the main astrocyte water channel AQP4 is up-regulated in reactive astrocytes and has a role in their migration and the formation of the glial scar during traumatic brain injury [72,73]. In cerebral edema, AQP4 has the main role in astrocyte swelling, which can lead to detrimental and even fatal outcomes in CNS diseases that are followed by edema, including ischemia, trauma, tumors, inflammation, and metabolic disturbances [74].

### 2.2. Types of Reactive Astrocytes

**Figure 2:**
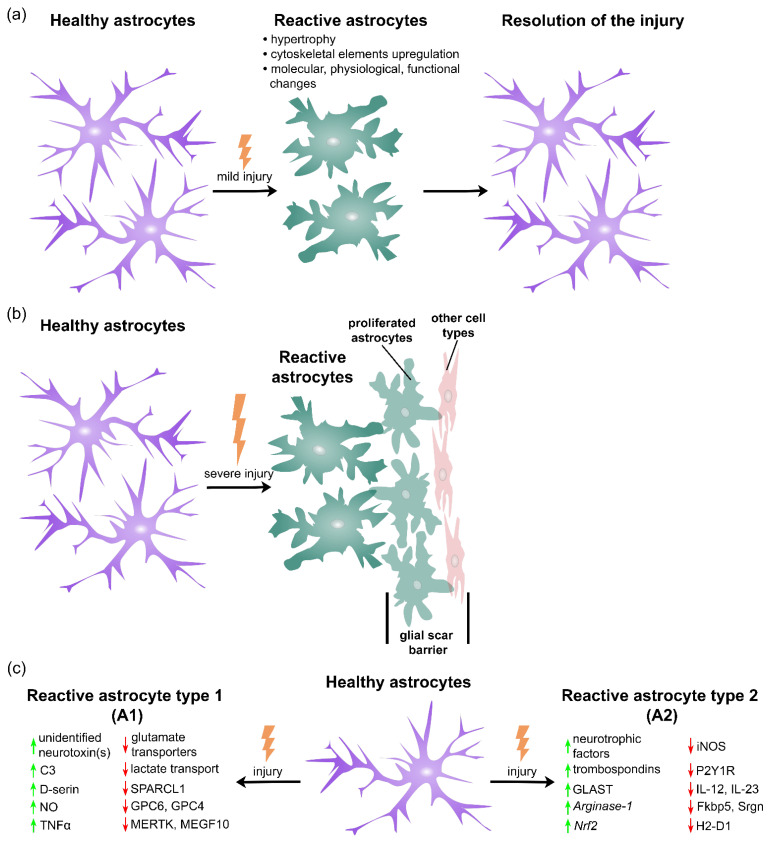
Types of reactive astrocytes. Based on the severity of the CNS injury, reactive astrocytes can be classified into two main subtypes: hypertrophic, and scar-forming reactive astrocytes. (**a**) As a result of mild CNS injuries (mild trauma, viral or bacterial infections) astrocytes become hypertrophic and go through variable levels of molecular, functional, and physiological changes. During mild injury, astrocytes do not proliferate, stay in their original positions, and they keep interacting with the same type of cells as in healthy CNSs. In this type of reactive astrogliosis, resolution of the injury is possible and there is no permanent tissue rearrangement. (**b**) As a result of severe injury, astrocytes proliferate and migrate to the site of injury where they form a glial scar. Glial scars comprise both newly proliferated astrocytes and other cell types. Severe injuries result in substantial permanent tissue reorganization and can inhibit axon regeneration at the site of injury. (**c**) Based on the pathological state that induced their reactivation, astrocytes can be classified as A1 or A2 astrocytes. A1 astrocytes can release pro-inflammatory factors such as unidentified neurotoxin(s), complement C3 (C3), D-serine, nitric oxide (NO), and tumor necrosis factor α (TNF-α) that can be detrimental for neuronal survival and recovery. They decrease lactate transportation; glutamate transporters; SPARC-like protein 1 (SPARCL1); glypicans: GPC4 and GPC6; MER Proto-Oncogene Tyrosine Kinase (MERTK); and Multiple EGF-like Domain 10 (MEGF10). The A2 type of reactive astrocytes promote CNS regeneration by secreting a subset of neurotrophic factors and thrombospondins which support the survival of neurons and the recovery of synapses, respectively. They also up-regulate the glutamate/aspartate transporter (GLAST) and, therefore, reduce extracellular glutamate preventing neurons from excitotoxicity. A2 astrocytes up-regulate genes involved in the anti-inflammatory response (*Arginase-1* and NF-E2-related factor 2 (*Nrf2*)), and down-regulate factors involved in stress response and inflammation: inducible nitric oxide synthase (iNOS); P2Y purinoceptor 1 (P2Y1R); interleukins IL-12 and IL-23; FKBP Prolyl Isomerase 5 (FKBP5); Serglycin (SRGN; histocompatibility 2 D region locus 1 (H2-D1); and Guanylate-Binding Protein 2 (GBP2).

There are different criteria for reactive astrocyte classification. One is based on the severity of the injury, while the other is based on the type of injury that induced the reactivation. According to the severity of the injury, reactive astrocytes can be classified in two main subtypes: hypertrophic reactive astrocytes and scar-forming reactive astrocytes [5,53]. Based on the pathological state that induced their reactivation, astrocytes can be classified as A1 and A2 reactive astrocytes [52,75,76] (Figure 2).

Hypertrophic reactive astrocytes are different from scar-forming reactive astrocytes in that they do not proliferate, stay in their original positions, keep interacting with the same type of cells as in healthy CNS, and keep their basic cell features and morphology. Depending on the severity of the CNS injury, this type of reactivated astrocyte undergoes variable degrees of cell hypertrophy and changes in gene expression. Hypertrophic reactive astrogliosis, also known as mild reactive astrogliosis, usually occurs as a result of mild trauma, viral or bacterial infections, or in areas that are not in close proximity to the focal CNS lesions [5,41,53]. This type of reactive astrogliosis does not lead to permanent tissue rearrangement, especially if the triggering factor is removed (Figure 2) [5,53].

In response to CNS injuries such as penetrating trauma, severe contusive trauma, ischemia, infection, autoimmune inflammation, toxin accumulation, NDs, or the disruption of the BBB, scar-forming reactive astrocytes migrate to the site of injury where they proliferate and form a border which separates the damaged tissue from the surrounding healthy neural tissue [39,41,53]. Newly proliferated astrocytes can derive either from mature astrocytes that are already present in the vicinity of the lesion, or they can derive from neural progenitors [41,53]. The physiological role of astrocyte scar borders, formed by these proliferative astrocytes and other cell types, is to prevent further damage and inflammation to the surrounding healthy neural tissue (Figure 2). On the other hand, this process is not reversible, and results in substantial permanent tissue reorganization and can lead to some detrimental effects on CNS recovery [5,41,53]. For example, glial scars have a negative effect on axon regeneration at the site of the injury [77,78].

Studies on mice using either lipopolysaccharide injection (generating neuroinflammation) or middle cerebral artery occlusion (generating focal cerebral ischemia) have shown that neuroinflammation induces the pro-inflammatory A1 phenotype, while ischemia induces the anti-inflammatory A2 phenotype of reactive astrocytes [52]. Transcriptome analysis that was performed on astrocytes isolated from mice showed that A1 astrocytes have detrimental effects on CNS recovery by up-regulating genes of the complement cascade that have a destructive effect on synapses. Particularly, A1 astrocytes can release pro-inflammatory factors such as unidentified neurotoxin(s), complement C3 (C3), D-serine, nitric oxide (NO), and TNF-α and, therefore, kill or inhibit the activity of a subset of neurons and oligodendrocytes in diseases [44,75]. As stated by Ding et al., A1 astrocytes also down-regulate glutamate transporters, GABA, trophic factors, genes involved in lactate transport, etc. [79]. A1 astrocytes lose the ability to induce synapse formation by down-regulating factors that are involved in synapse formation (SPARC-like protein 1 (SPARCL1)) and glypicans (GPC6 and GPC4) [76,79]. They also lose the ability of synaptic phagocytosis and the clearance of myelin debris by down-regulating phagocytic receptors MER Proto-Oncogene Tyrosine Kinase (MERTK) and Multiple EGF-like Domain 10 (MEGF10) [76]. It has been shown that the A1 phenotype is regulated by NF-κB [80]. On the other hand, the A2 type of reactive astrocytes promote CNS regeneration by secreting a subset of neurotrophic factors and thrombospondins which support the survival of neurons and the recovery of synapses, respectively [44]. Neal et al. found that the A2 type of mouse primary reactive astrocytes up-regulate glutamate transporter GLAST expression and, therefore, reduces extracellular glutamate levels, preventing neuronal degeneration due to excitotoxicity [81]. They also showed that treatment of astrocytes with the chemokine-like signaling protein Prokineticin-2 (PK2) shifts them toward the A2 phenotype by increasing the anti-oxidant genes of *Arginase*-1 and NF-E2-related factor 2 (*Nrf*2), and decreasing the expression of inducible nitric oxide synthase (iNOS) responsible for the exacerbation of the pro-inflammatory response [81]. It has been shown that A2 astrocytes also down-regulate some of the factors involved in stress response and inflammation, including: P2Y purinoceptor 1 (P2Y1R), IL-12, IL-23, FKBP Prolyl Isomerase 5 (FKBP5), Serglycin (SRGN), histocompatibility 2 D region locus 1 (H2-D1), and Guanylate-Binding Protein 2 (GBP2) [79]. There are strong indications that the A2 phenotype is regulated by STAT3 [75]. Both A1 and A2 reactive astrocyte phenotypes are found during brain aging, as well as in different pathological states including injuries, infection, and different NDs [75]. Although classification to A1 and A2 astrocytes has been adopted in multiple studies [52,75,76], this simple binary division of reactive astrocytes to two distinct good/bad or neurotoxic/neuroprotective reactive states has its disadvantages, because it fails to capture their diversity across CNS diseases [82]. The improved explanation, which was given by Escartin et al., is that at any given time-point during pathology progression, there is a continuous spectrum of reactive astrocyte phenotypes which coexist in the brain [82]. Additionally, the gain of protective functions and the loss of beneficial functions of reactive astrocytes during disease can happen simultaneously. The balance of lost/gained functions as well as the abundance of different astrocyte subpopulations is what determines the final outcome of the disease [44,82].

### 2.3. The Function of Reactive Astrogliosis in the Brain Pathogenesis

#### 2.3.1. Ischemia

The main adverse effect of brain ischemia is the loss of brain energy supply and oxygen [83]. Stroke, the most common type of focal ischemia, is caused by brain artery occlusion, leading to neuronal loss in the ischemic core and glial scar formation in the penumbral region around the core [84]. Ischemia is a serious brain condition that can lead to severe brain damage, disability, or even death. In the past, research focus has been directed toward neurons, since they are more sensitive to ischemia than astrocytes. Today, it is known that astrocytes have important and diverse roles in this pathology, which makes them promising therapeutic targets for the treatment of brain ischemia [85,86]. During ischemia, astrocytes undergo changes typical for reactivation, including cellular hypertrophy, increased proliferation and glial scar formation, gene expression changes and the increased expression of intermediate filaments (GFAP, VIMENTIN, NESTIN). They can exert both beneficial and detrimental roles during brain ischemia [87,88]. They aggravate inflammatory responses by secreting cytokines. A study by Li et al. has shown that, during ischemia, reactive astrocytes secrete IL15, which specifically activates the cytotoxic effects of natural killer (NK) cells and CD8+ T lymphocytes, which leads to tissue inflammation and damage after ischemic injury [89]. On the other hand, astrocytes also exert beneficial functions by producing several angiogenic and neurotrophic factors that are important for neuronal survival and regeneration after stroke [88,90]. The loss of the main energy substrates—glucose and oxygen—during ischemia decreases the synthesis of cellular ATP [86]. The loss of cellular ATP leads to inhibition in the functioning of Na^+^-K^+^ ATPase, which subsequently leads to the loss of ionic gradients and membrane depolarization in both astrocytes and neurons. This chain of events causes a release of glutamate into the extracellular space resulting in the death of neurons, and potentially astrocytes, due to excitotoxicity. It has been shown that selenium nanoparticles suppress ischemia-induced neural damage by stimulating the release of ATP and lactate from astrocytes into extracellular medium. These energy substrates prevent neuronal death caused by ischemia/reoxygenation [91]. Intercellular signaling among the neurons, glial and brain endothelial cells after ischemic stroke has been considered to mediate angiogenesis [92], and is considered as an important therapeutic strategy in the stroke [93].

**Figure 3:**
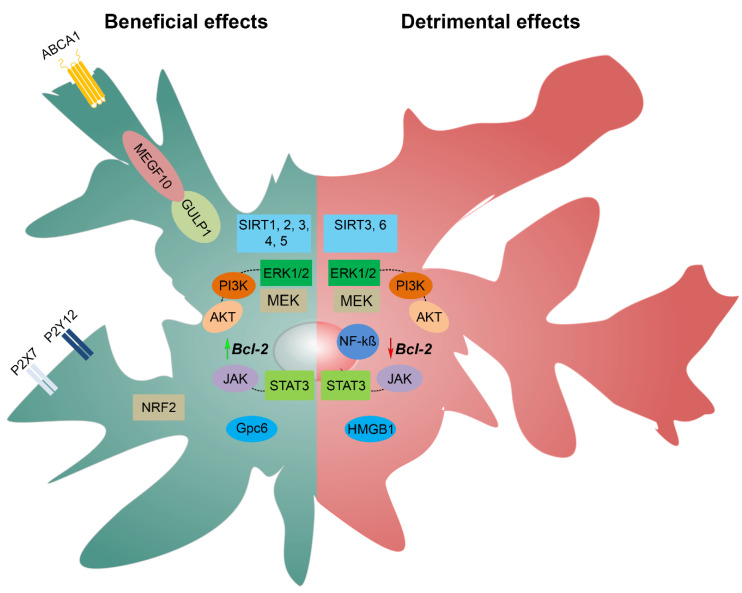
Simplified schematic representation of the major signaling pathways involved in astrocytes upon ischemia, displaying beneficial and/or detrimental effects in CNS pathology. Sirtuin 1, 2, 4 and 5 (SIRT1, SIRT2, SIRT4, and SIRT5, respectively) are shown to protect the brain. Sirtuin 3 (SIRT3) is largely neuroprotective in ischemic stroke, but it has been also shown that it can lead to the mitochondrial accumulation of the pro-apoptotic lipid molecule. Sirtuin 6 (SIRT6) leads to necrotic cell death, while the consequence of sirtuin 7 (SIRT7) up-regulation is still unknown. The purinergic pathway in reactivated astrocytes mainly acts through Purinergic P2X Receptor 7 (P2X7) and Purinergic Receptor P2Y G-Protein Coupled 12 (P2Y12) receptors showing beneficial effects. Multiple EGF-like Domain 10 (MEGF10) and GULP PTB Containing Engulfment Adaptor 1 (GULP1) as phagocytic pathways are up-regulated together with ATP Binding Cassette Subfamily A Member 1 (ABCA1) and contribute to the remodeling of damaged tissues. NF-E2-related factor 2 (NRF2) also shows a neuroprotective role, while the NF-kß signaling pathway contributes to the progression of ischemia. MAPK/ERK, PI3K/AKT and JAK/STAT3 signaling pathways can aggravate or alleviate neuronal damage upon ischemia. The MAPK/ERK pathway shows pro-apoptotic roles in astrocytes upon ischemia, while the activation of this pathway is able to mediate post-ischemic astrocyte proliferation and survival trough changes in Bcl-2-B-cell lymphoma 2 (Bcl-2) gene expression. PI3K/AKT can either induce neuronal survival or neuronal death through changes in Bcl-2 gene expression. The inhibition of the JAK2/STAT3 signaling pathway can reduce the expression of High-Mobility Group Box 1 (HMGB1) in brain tissue after cerebral ischemia, which further reduces the inflammatory response, while the STAT3 pathway can target glypican 6 (Gpc6) in astrocytes towards neuroprotective phenotypes and neural regeneration.

Several signaling pathways, displaying beneficial and/or detrimental effects in CNS pathology, are implicated in the reactivation of astrocytes after ischemia (Figure 3). Purinergic, NRF2, and MEGF10/GULP PTB Containing Engulfment Adaptor 1 (GULP1) signaling pathways show neuroprotective roles by supporting neuronal function and the survival and remodeling of damaged tissue [94,95,96,97]. On the other hand, NF-kß signaling pathway contributes to neuronal death and increased inflammation [98], while sirtuin (SIRT), Phosphoinositide 3-kinase (PI3K)/AKT serine/threonine kinase 1 (AKT), MAPK/extracellular signal-regulated kinase (ERK), and the JAK/STAT3 signaling pathways can aggravate or alleviate neuronal damage upon ischemia [96,99,100,101,102,103]. However, signaling pathways in the brain, in the terms of ischemia, represent a dynamic field of research which still requires further elucidation. In addition, the distinction of signaling pathways that trigger beneficial responses upon ischemia is challenging due to the dual role of astrocytes [85]. Therefore, a better understanding of which signaling pathways control which aspects of reactive astrogliosis is important for the future design of therapeutic strategies for CNS pathologies [104].

Cerebral edema is a key pathological finding in ischemic stroke resulting from the dysregulation of water homeostasis in the CNS. It plays a major role in stroke-associated morbidity and mortality. Water balance in the brain is tightly regulated, primarily by the AQP4 channels, which are mainly expressed in perivascular astrocytic endfeet and have a central role in the formation and clearance of cerebral edema [105,106]. Although targeting these plasma membrane water channel proteins could be a useful therapeutic approach for treating brain edema and other conditions associated with disrupted water and solute homeostasis, aquaporin drug discovery has made little progress. This is possibly due to the non-druggability of the aquaporin pore, or the lack of reliable in vitro assays suitable for screening and validating the pharmacological regulation of aquaporin function [107]. For instance, the *Xenopus laevis* oocyte assay is considered as the gold-standard assay in the field, even though it suffers from a lack of inter-laboratory reproducibility [107]. The biggest disadvantage of this and other shrinkage/swelling assays commonly used for the functional analyses of aquaporin water transport [108] is their incompatibility with high-throughput screening formats. By high-throughput analyses, large-scale compound libraries containing thousands of molecules with potential therapeutic relevance can quickly be screened in an accelerated and cost-effective way, but there are no currently validated high-throughput assays of aquaporin function [107]. An alternative to blocking the AQP4 water pore has recently been suggested by targeting AQP4 membrane trafficking and subcellular localization. In particular, calmodulin has been shown to play a central role in the subcellular relocation of AQP4, and its pharmacological inhibition in rat models of traumatic spinal cord and brain injury led to reduced edema and enhanced functional recovery [105]. This mechanism of action has recently been confirmed by the work of Sylvain et al. [106] that has demonstrated that targeting AQP4 is a viable therapeutic target in photothrombotic stroke models. They have also shown a link to brain energy metabolism, as indicated by the increase in glycogen levels [106].

#### 2.3.2. Neurodegenerative diseases

Alzheimer’s disease (AD), amyotrophic lateral sclerosis (ALS), Parkinson’s disease (PD), and Huntington’s disease (HD) are NDs that typically occur in late adulthood. One of the pathological characteristics of these neurodegenerations is neuroinflammation [109,110,111,112]. Inflammation in the CNS can have beneficial effects, such as fast and localized reactions to the injury [113]. However, in these NDs, neuroinflammation is widespread, prolonged, and can become detrimental [109].

Recently, a newly proposed concept of inflammaging was introduced as one of the causes contributing the development of NDs [114]. Inflammaging is considered to be chronic, sterile, low-grade inflammation that occurs during aging [114], with its major characteristic being chronic activation of the innate immune system [115]. Various cellular and molecular mechanisms (e.g., cellular senescence, immunosenescence, mitochondrial dysfunction, defective autophagy, metaflammation, and gut microbiota dysbiosis) are involved in this process [116]. Inflammaging has been recognized as one of the factors contributing to age-related cerebral small vessel disease, which is most prevalent among elderly people and contributes to a high global disease burden of stroke and vascular dementia [117].

The complex interplay between inflammatory mediators, ageing, genetic back-ground, and environmental factors may ultimately regulate the outcome of acute CNS injury and the progression of chronic neurodegeneration, and be critical for the development of effective therapies for CNS diseases [118]. Astrocyte reactivity is generally associated with microglial reactivity and leukocyte recruitment. Depending on the timing and context, astrocyte reactivation may exacerbate inflammatory reactions and tissue damage, or promote immunosuppression and tissue repair [119]. Reactive astrocytes and microglia produce inflammatory molecules and are involved in shaping the inflammatory response to CNS injuries. Inflammatory mediators secreted by pro-inflammatory microglia may activate pro-inflammatory astrocyte phenotype and induce a secondary inflammatory response [76,119,120,121]. Previous studies have shown that the ablation of reactive astrocytes enables increased leukocyte infiltration into injured CNS parenchyma, contributing to the pathogenic mechanisms for inflammatory disturbance in the CNS [122]. In vivo, astrocytes were exposed simultaneously to a plethora of stimuli as the result of a complex network of intracellular activatiors to activate distinct intracellular signaling pathways during neuroinflammation [119].

A recent study examining reactive astrocyte signatures in neuroinflammation showed the presence of A1 astrocyte phenotype activated by microglia-derived IL-1α, TNF, and complement component 1q (C1q) in the different brain regions of AD, PD, ALS, MS, and HD patients and animal models [76]. Reactive A1 astrocytes lose basic homeostatic astrocytic functions, gain toxic function, and release ROS and other toxic metabolites, and are suggested to contribute the death of neurons and the progression of degeneration [76]. Astrocytes may have multiple simultaneous reactive profiles, but with a continuous spectrum, emphasizing the importance of the investigation of the heterogeneity of reactive astrocytes [44]. Recent studies have shown that activated endothelial cells cause increase in the expression of C3 and other components of the complement system in astrocytes, similar to the microglia-induced A1 astrocytic phenotype [123]. RNAseq showed that these astrocytes have different molecular signatures than astrocytes activated by microglia and significant increase in the expression of extracellular matrix genes. They exhibit a neurotoxic gain of function, and were associated with vascular amyloid deposits, but not amyloid β plaques, in the parenchyma of AD patients and a mouse model [123].

Crosstalk between astrocytes and neurons is essential for the proper functioning of neurons, as well as neuronal defense against ROS and other stressors. The reactive astrocyte phenotype A1 leads to neuronal loss by promoting inflammation via the NF-kB pathway, which loses the ability to protect neurons and control synaptogenesis [124,125], while A2 astrocytes promote neuronal survival via the JAK/STAT3 signaling pathway by up-regulating neurotrophic factors [126].

Whether the effects of astrogliosis are beneficial or detrimental depends on the time-point in disease progression, specific disease, and different stimuli from the microenvironment, such as microglia [120], and other cell types [127]. The exact roles of reactive astrocytes are hard to define, as appropriate models are missing [128]. Although the complex interplay between cell types is present in NDs, in this review, we will predominately focus on some of the characteristics of reactive astrocytes and their roles in coordinated and complex processes of neuroinflammation, as a better understanding of the roles of astrocytes in NDs is essential for developing effective therapies and possible targets in therapeutic approaches.

Alzheimer’s disease is characterized with severe memory and cognitive impairment and dementia [129]. Extracellular amyloid β (Aβ) plaques and intracellular hyperphosphorylated and cleaved forms of the microtubule-associated protein tau, forming neurofibrillary tangles (NFTs), are pathological features of this disease [40,130]. Besides the development of these lesions, AD is associated with the disappearance of synapses, dendritic branches, and neurons [131]. The reactivation of astrocytes was detected in AD patients as well as in animal models before the presence of significant amyloidosis and neurodegeneration [132,133]. Reactive gliosis and hypertrophic GFAP+ astrocytes were observed in the vicinity of Aβ plaques in the cerebral cortex of an AD transgenic model [134]. Although one study showed a proliferative response of only a small portion of GFAP+ and/or S100β+ reactive astrocytes in a transgenic model of AD [134], the ablation of proliferative astrocytes exacerbated the disease [135]. Increased levels of non-aggregated forms of Aβ, significant neuronal and synaptic loss in the hippocampus, and increases in pro-inflammatory cytokine production and the activation of NF-kB were observed after the ablation of reactive astrocytes in an AD model [135], implying their role in amyloid peptide clearance and the control of neuroinflammation in AD progression. Gene expression analysis showed that genes up-regulated in astrocytes in Aβ and tau models of AD were associated with cytokine and inflammatory responses and protein degradation [136]. One of the up-regulated genes was the transcription factor Nrf2, which is involved in inflammatory and oxidative stress responses [137,138], potentially presenting an adaptive–protective response to Aβ reactive astrocytes [136]. Astrocyte-specific Nrf2 expression induced a reactive phenotype which reduced Aß deposition and phospho-tau accumulation, and rescued transcriptional deregulation, cellular pathology, neurodegeneration, and behavioral/cognitive deficits [136]. The heterogeneity of the reactive states of astrocytes might be due to the severity of stimuli inducing reactivation [128]. The relationship between reactive astrocytes and neurodegeneration in AD was demonstrated through the induction of severe astrogliosis which led to neurodegeneration [128]. Aβ evoked astroglial NF-κB activation and the release of C3 which, through the neuronal C3a receptor and intraneuronal calcium, increased synaptic excitation and disrupted dendritic morphology, the combination of which led to network dysfunction [124].

Amyotrophic lateral sclerosis is characterized by the paralysis, atrophy and motor impairment pathologically characterized by the death of the upper and lower motor neurons. Although most of the cases are of unknown etiology, mutations in *SOD1*, *C9ORF72*, *FUS* and *TDP43* genes are known to cause the disease. Misfolded protein aggregations and inclusions, impaired RNA processing, and neuroinflammation are implicated in the potential mechanisms of the disease [139,140]. The importance of glial cells has been shown for the disease’s onset and progression [141]. Reactive astrocytes expressing A1 phenotypes were observed in ALS patients in regions affected by the disease [76]. Studies showed that ALS astrocytes in coculture with neurons caused the death of, specifically, α motor neurons by the secreted toxic soluble factors [76,142,143]. Gene expression analyses in astrocytes derived from ALS patients showed the up-regulation of genes for chemokines and pro-inflammatory cytokines and the NF-κB signaling complex as a major interactor [142]. The specific inhibition of NF-κB in astrocytes was not able to prevent the death of motor neurons, implying the activation of other signaling pathways [144] and the complex interplay between glial cells, as speculated in ALS [76,141]. The knockout of IL-1α, TNFα, and C1q in the ALS model reduced astrocyte reactivation and motor neuron death and prolonged life span in an ALS animal model [145]. Reactive astrocytes were shown to exhibit the deregulation of homeostatic functions in ALS. The down-regulation of glutamate transporter EAAT2 expression in reactive astrocytes in ALS highlighted the role of glutamate excitotoxicity in mechanisms of neurodegeneration [146,147]. A study on astrocytes, differentiated from induced pluripotent stem cells (iPSCs) from ALS patients, showed the reduced expression of Kir4.1 and the up-regulation of GFAP, implying the importance of the loss of homeostatic functions of astrocytes in the progression of neuroinflammation in neurodegeneration [148]. Changes in reactive astrocytes were shown to be detrimental, not only to motor neurons in ALS, but to the mature oligodendrocytes as well [76], emphasizing the importance of astrocyte reactivation in ALS pathology.

Parkinson’s disease is marked by severe motor disability and cognitive impairment [149]. Pathologically, it is characterized by the loss of dopaminergic neurons in the substantia nigra [150], neuroinflammation [151], and the presence of α-synuclein-positive cytoplasmic inclusions [152]. Mutations in different genes implicated in PD pathology were shown to be involved in the regulation of astrocytic reactivation in response to inflammatory stimuli [152]. Studies have shown that reactive PD astrocytes exhibited a more profound response to the exposure to the inflammatory stimuli compared to the control astrocytes [153]. Sonninen et al. demonstrated that reactivation using TNF-α and IL-1β caused the increased secretion of IL-6 and CCL5 (RANTES) and the up-regulated expression of GFAP and lipocalin-2 (LCN-2) in PD astrocytes to a higher extent, compared to the changes observed in the control astrocytes. LCN-2 was demonstrated to be a promoter of the pro-inflammatory reactivation of astrocytes [154,155], suggesting the pathogenic role of reactive astrocytes in PD [153]. Reactive astrocytes in PD showed the redistribution of AQP4 expression, impairing blood vessel shielding [156], as well as a reduced expression of EAAT2 [156,157], indicating that some of essential astroglial functions are altered in this disease.

Huntington’s disease is characterized by its progressive motor, cognitive and psychiatric symptoms [158]. The death of neurons in the striatum and other brain regions, the aggregation of mutant huntingtin, and the presence of reactive astrocytes [159] are considered to be pathophysiological hallmarks of the disease. Reactive astrocytes in the striatum of HD patients are shown to display hypertrophic morphology and the increased expression of GFAP [160]. Increases in GFAP immunoreactivity were observed in the late stage of the disease in an HD animal model [158]. Gene expression analysis of astrocytes from HD models and patients implied the existence of genes with expressions linked with the expression of the astrocyte mutant huntingtin protein [161]. Contrary to a previously published study [76], Diaz-Castro et al. [161] suggested that astrocytes in HD lacked the A1 phenotype, considered to contribute to neuronal death in neurodegeneration, but instead showed changes in physiological functions. The study showed the decreased expression of Kir4.1, altered glutamate clearance, and altered K^+^ homeostasis in HD astrocytes, which are all suggested to contribute to the neuronal hyperexcitability in HD animal models [158]. Other studies have shown significant increases in the subsets of A1, A2 and pan-astrocytic genes, among them GFAP, CD44, CXCL10, C3, and CD14 [162]. The activation of the JAK2/STAT3 pathway was observed in the putamen of HD patients and the selective activation of this pathway in astrocytes reduced the number of aggregates in neurons and striatal atrophy, and increased glutamate levels [159]. The inhibition of the JAK2/STAT3 signaling pathway in astrocytes prevented astrocyte reactivity, reduced microglia activation, and increased the number of huntingtin aggregates, but did not influence neuronal death [163]. Transcriptomics showed that JAK2/STAT3 increased intrinsic proteolytic capacity through the lysosomal and ubiquitin/proteasome degradation system, implying a beneficial response to reactive astrocytes, potentially improving disease outcome [159].

Multiple sclerosis is a chronic inflammatory disease characterized with episodes of neurological disability which are partially or fully reversible [164]. In MS, a disease with typical onset in the middle life, neuroinflammation is considered to lead the disease progression while neurodegeneration occurs subsequently [165]. Pathological hallmarks are lesions in the white matter presented as areas of demyelination, inflammation, and glia reaction [166]. Acutely demyelinating lesions prograde into chronic active lesions, characterized by complete demyelination, astroglial scarring in the lesion core, and inflammatory cells at the lesion periphery [167]. Reactive hypertrophic astrocytes are localized at the rim of the demyelinating lesions, and are shown to phagocyte myelin [168,169]. It has been shown that the process of myelin phagocytosis caused the activation of astrocyte NF-κB signaling and chemokine secretion CCL10, CCL5, and CXCL3 in order to recruit inflammatory cells, but the expression of pro- and anti-inflammatory cytokines was not observed [168]. Astrocyte activation in MS is suggested to have a role in the initialization of myelin clearance as the necessary reaction for microglia recruitment and myelin removal and the initiation of remyelination [170].

## 3. Morphological and Molecular Features of Senescent Astrocytes

**Figure 4:**
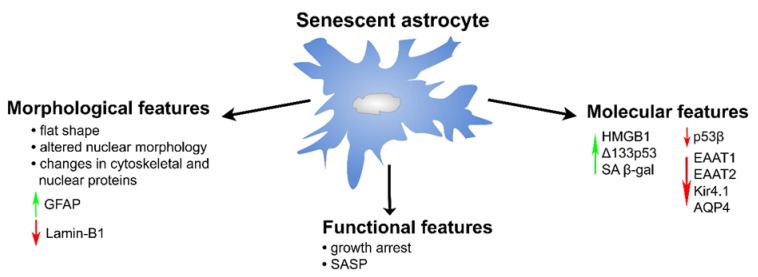
Major morphological molecular and functional features of senescent astrocytes. Senescent astrocytes display an enlarged, flattened cell shape with altered nuclear morphology, accompanied by changes in cytoskeletal and nuclear protein expression, in particular, an increase in glial fibrillary acidic protein (GFAP) and a decrease in Lamin-B1. Senescent astrocytes show the diminished expression of Δ133p53 and increased High-Mobility Group Box 1 (HMGB1) protein, p53β, and senescence-associated β-galactosidase (SA ß-gal) activity. The expression of glutamate transporters (EAAT1/EAAT2), potassium channel (Kir4.1), as well as water transporter AQP4 are all down-regulated. Complex morphological and molecular alterations provide predispositions for the adoption of the main senescence characteristics: growth arrest and the senescence-associated secretory phenotype (SASP) secretome.

Cellular senescence was originally identified as a stable cell cycle arrest of cultured human fibroblasts [171]. It was later found that senescent cells can accumulate in various tissues and organs where they can display different physiological and pathological functions [172]. Senescence is an irreversible physiological mechanism that can be triggered in response to numerous internal and external stressors [173]. Due to their proliferative capacity, astrocytes also undergo cellular senescence that Cohen et al., named as “astrosenescence” [174]. Numerous studies show that astrocytes can display senescence after exhausted replication, oxidative stress, proteasome inhibition, high glucose, HIV infection, X radiation, hydrogen peroxide, or in the course of aging [175,176,177]. In contrast to reactive astrogliosis, the cellular senescence of astrocytes is a permanent state [174]. Even though senescent astrocytes exhibit both morphological and molecular changes that are classical hallmarks of cellular senescence in general (Figure 4), here we focused only on those characteristics of senescent astrocytes that have been experimentally validated.

Morphological alterations observed in senescent astrocytes are mainly associated with changes in cell shape, the reduced expression of nuclear lamina protein Lamin-B1, and intermediate filament reorganization (Figure 4). After exposure to oxidative stress, primary cultures of both mouse and human astrocytes displayed an enlarged, flattened, and vacuolized morphology typical for senescent cells [178]. Matias et al. showed on senescent astrocytes in vitro that defective nuclear morphology is represented by an increased incidence of invaginated nuclei and the loss of nuclear circularity that is accompanied by the loss of Lamin-B1 [179]. This finding is not surprising since Lamin B1 is a major component of nuclear lamina, and altered nuclear morphology in senescent cells due to the loss of this lamina has also been previously reported [180]. Similarly to reactive astrocytes, senescent astrocytes are characterized by increased GFAP expression [181]. It is noteworthy to mention that GFAP is important for maintaining the mechanical strength and shape of astrocytes [182].

Besides morphological changes, senescent astrocytes display complex molecular alterations and distinct phenotypic alterations followed by dramatic changes in gene expression (Figure 4) [183]. Complex morphological alterations of senescent cells, together with molecular and phenotypic alterations, make predispositions for the adoption of the main senescence characteristics: growth arrest and the senescence-associated secretory phenotype (SASP) secretome (Figure 4) [183].

Unlike quiescent cells where cell cycle arrest happens in G0, senescence triggers the permanent cell cycle arrest in the G1/G2 phase of the cell cycle [183]. In senescent astrocytes, there is only a modest amount of telomere shortening, which is a hallmark of senescent cells [184]. Unlike in fibroblasts, human telomerase reverse transcriptase (hTERT) is not able to extend the replicative life span of astrocytes, indicating that this permanent cell cycle arrest in astrocytes, known as replicative senescence, is p53-dependent and telomere-independent [184]. p53, the protein known as the “guardian of a genome” with versatile functions is also known as a hallmark of senescence, having a pivotal role in this process [185]. There are two isoforms of p53 protein in astrocytes, ∆133p53 and p53β, and senescent astrocytes showed the diminished expression of Δ133p53 and increased p53β (Figure 4) [186]. Turnquist et al. also showed that the reconstitution of the Δ133p53 isoform in human astrocytes had a protective role from radiation-induced senescence [187]. These two isoforms of p53 are considered as endogenous regulatory mechanisms for p53-mediated replicative senescence [188]. Senescent astrocytes display the increased expression of one of the first downstream targets of p53, cell cycle regulator p21^waf1^, which further inhibits cyclin D-dependent kinase CDK2 activity resulting in cell cycle arrest [176]. This signaling pathway, together with the p16^ink4A^/proteins of the retinoblastoma family (pRB) pathway, plays a central role in the regulation of cell cycle arrest in senescent astrocytes [178,184,189]. Therefore, astrocytes possess two distinct inducible growth arrest pathways that can have redundant function in promoting the inhibition of proteins involved in cell cycle progression [189]. Both these pathways are complex and include alterations in different sets of genes [183]. p53 and p21^waf1^ are crucial players in senescence, and it has been suggested that the p53/p21^waf1^ pathway is important for the initiation of senescence, while the pathway involving p16 and the pRb family is important for the maintenance of senescence [190].

SASP is characterized by the increased secretion of chemokines, growth factors, proteases, and inflammatory cytokines leading to the induction of inflammation [191]. Interestingly, as proposed by Bhat et al., several SASP components that are altered in other cell types show no obvious changes in senescent astrocytes [192]. In addition, alterations in SASP are stimulus-dependent [176]. Although the composition of SASP secretomes is variable, one of the most consistent SASP components and common feature of senescent astrocytes is the induced pro-inflammatory SASP cytokine IL-6 [176]. The expression of IL-6 in senescent astrocytes is under the direct regulation of the Wnt/ß-catenin and p38 MAPK pathways [193]. Besides IL-6, interleukins IL-8 and IL-1ß and TNF-ɑ are other pro-inflammatory cytokines often increased in senescent astrocytes, while IL-2, IL-3 and IL-4 show no or little induction [177,186,194,195]. Besides interleukins, other components of SASP increased in senescent astrocytes are metalloproteinases MMP-1 and MMP-3, and chemokine CXCL-1 [177,194,195]. SASP component induction is under the control of the HMGB1 protein, a member of the highly conserved non-histone DNA-binding HMG protein family. Nuclear translocation and the secretion of HMGB1 is considered a hallmark of cells entering senescence (Figure 4) [196]. The secretion of HMGB1 was also detected in senescent astrocytes [177]. Shang et al. showed that the inflammatory cytokine IL-1β was able to induce cellular senescence in astrocytes through the activation of the SASP pathway (IL-6, IL-8 and MMP-3), raising an interesting question as to whether inflammatory factors lead to cellular senescence in the brain or whether cellular senescence leads to the rise of inflammatory factors [195].

The most used biomarker and the most used techniques for the detection of senescent cells is the senescence-associated β-galactosidase (SA-β-gal) (Figure 4) [197]. Senescent astrocytes show SA ß-gal activity both in vitro and in vivo as the result of enhanced lysosomal content [174,178,184,192]. 

Since miRNAs are often altered during aging, it is suggested that miRNAs are potential sensors of cellular senescence [198]. Some miRNAs involved in the regulation of the main processes altered in senescence are referred to as senescence-associated miRNAs (SA-miRNAs) [199]. Mainly, miRNAs influence cellular senescence through the modulation of the expression of senescence regulatory proteins, particularly the protein components of the p53/p21^waf1^ and p16^ink4A^/pRB pathways and SASP [200]. While the roles of miRNAs have received a lot of attention in the context of reactive astrocytes, there are almost no data about their roles in the initiation or maintenance of senescence. Even though it was shown that miR-29 plays important roles in aging, no effect of this miRNA was observed on the cellular senescence of astrocytes [201]. We have recently shown that the down-regulation of miR-21 in NT2/D1-derived astrocytes was able to induce growth arrest and premature cellular senescence [202]. Moreover, our in silico analysis predicted many of the genes, previously shown to be up-regulated in astrocytes with irradiation-induced senescence, as potential miR-21 targets. Interestingly, these putative miR-21 targets also include the genes related to SASP [202]. To the best of our knowledge, this is the first study that implicates miRNAs in the regulation of senescence in astrocytes. It looks like this is a wide-open field, and we suggest that future studies should include comprehensive research on the role of miRNAs in astrocyte senescence. Understanding the molecular mechanisms by which miRNAs regulate senescence is of particular interest for developing novel diagnostic and therapeutic opportunities for the treatment of diseases that are associated with senescent astrocytes [200].

The expression of genes coding for proteins important for glutamate and potassium transport (EAAT1/EAAT2 and Kir4.1), as well as water transporter AQP4, was down-regulated in senescent astrocytes, and this down-regulation was persistent (Figure 4) [177]. Importantly, it was shown that alteration in the expression of these genes negatively affected the glutamate uptake of astrocytes and induced the accumulation of glutamate released by neurons in synapses, which further induced glutamate toxicity leading to the death of neurons [177]. These morphological and molecular alterations adopted as senescence features change the astrocyte physiology essential for neuronal support and, therefore, senescent astrocytes impact neuron functionality leading to their degradation. Besides their negative effects on neurons, senescent astrocytes also induce changes in the brain, such as impaired synaptic plasticity, neural stem cell loss, and BBB dysfunction [176]. All these alterations in brain induced by senescent astrocytes might lead to age-related NDs.

### Senescent Astrocytes and Age-Related Neurodegenerative Diseases

**Figure 5:**
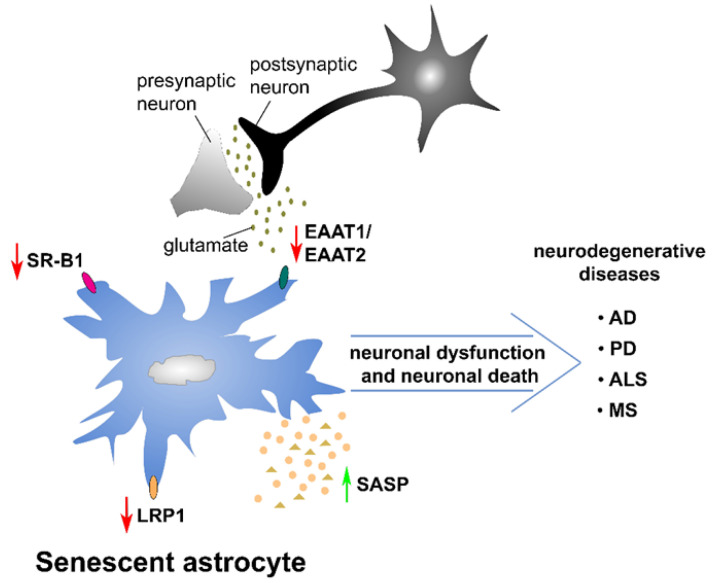
Characteristics of senescent astrocytes in the pathology of neurodegenerative diseases. Senescence-associated secretory phenotype (SASP) factors released from senescent astrocytes contribute to the chronic inflammatory environment. Due to the down-regulation of the expression of glutamate transporters, senescent astrocytes lose their ability to clear glutamate released from neurons, which results in the accumulation of extracellular glutamate, leading to excitotoxicity. The reduced expression of scavenger receptor class B type 1 (SR-B1) and low-density lipoprotein receptor-related protein-1 (LRP1) results in an altered capacity of senescent astrocytes to uptake and degrade Aβ. Together, these alterations lead to neuronal dysfunction and neuronal death, which results in the development of NDs, including Alzheimer’s disease (AD), Parkinson disease (PD), amyotrophic lateral sclerosis (ALS), and multiple sclerosis (MS).

Aging is a physiological process determined by genetics and influenced by various environmental factors [203]. A hallmark of aging is the accumulation of senescent cells that are unable to replace damaged cells that naturally accumulate over time [204]. In addition, senescence is considered as a primary inducing factor of age-related disorders such as NDs. Even though the role of cellular senescence in age-related diseases remains poorly understood, due to the alterations in a wide spectra of morphological and molecular features leading to the dysfunction of physiological functions, senescent astrocytes are considered as important players in NDs (Figure 5) [174]. The number of senescent astrocytes increases in human brain tissue during normal aging [205]. Senescent astrocytes were also identified in postmortem brain tissue from AD, PD, and ALS patients [192,206,207]. It is evident that senescent astrocytes are implicated in the pathology of AD, PD, and other NDs, but the underlying mechanisms are still incompletely understood. It is suggested that the main mechanism through which senescent astrocytes contribute to age-related diseases is inflammation [208,209]. This is particularly achieved through the altered secretion and expression of SASP components which create a proinflammatory microenvironment to neighboring cells [192,210]. Besides inflammation, alteration in glutamate uptake is another feature that contributes to the involvement of senescent astrocytes in NDs. Due to reduced glutamate transporter expression (EAAT1/EAAT2), senescent astrocytes lost their ability for glutamate uptake, further leading to neuronal death [177]. This deregulated glutamate metabolism in senescent astrocytes is a common feature of NDs (Figure 5) [207]. It was also shown that senescent astrocytes decreased neuronal mitochondrial membrane potential and altered neuronal mitochondria functionality after being cocultured with neurons [175]. The authors suggested that this could be due to the fact that senescent astrocytes either lost their ability to secrete factors required for the proper function of neurons or that they gained neurotoxic effect through the secretion of SASP factors [175]. It is more than evident that through the adoption of senescent phenotypes, astrocytes become functionally deregulated and lose their roles as vital protectors of neurons, but the precise mechanism of their implications in the etiology of CNS pathologies remains to be elucidated.

Bhat et al. were first to propose that the accumulation of senescent astrocytes may be one of the age-related risk factors for sporadic AD [192]. It was later suggested that SASP components contribute to major pathological mechanisms of AD such as Aβ accumulation, tau hyperphosphorylation, and the deposition of NFTs [176]. In fact, extracellular deposits of Aβ and NFTs consisting of aggregates of hyperphosphorylated tau protein are major components of senile plaques that represent cardinal features of AD [211]. It is widely accepted that the major cause of AD development is amyloid cascade hypothesis, which posits Aβ as the cause of AD. This further triggers the formation of NFTs, neuronal cell loss, vascular damage, and dementia [212]. In one study, senescent astrocytes were observed in the brain region surrounding Aβ plaque, and it was suggested that Aβ pathology is closely linked with senescence-associated inflammation [192]. The expressions of the astrocyte receptor low-density lipoprotein receptor-related protein-1 (LRP1) and scavenger receptor class B type 1 (SR-B1) decrease with aging (Figure 5) [213]. The reduced expression of these astrocyte receptors that are known to be involved in the uptake and clearance of Aβ can provide the explanation for the altered capacity of senescent astrocytes to uptake and degrade Aβ [176]. It is therefore suggested that the altered physiology of senescent astrocytes has an important role in Aβ accumulation, but the mechanisms underlying this process still need to be elucidated. Tau accumulation, as another hallmark of AD, was also discovered in astrocytes in the dentate gyrus of patients suffering from AD [214]. In AD and related tauopathies, tau, which originally maintains the stability of microtubules, is hyperphosphorylated, leading to the accumulation of insoluble filaments in NFTs [215]. It has been shown that the clearance of senescent astrocytes from a mouse model of tau-dependent ND prevented the degeneration of cortical and hippocampal neurons and preserved cognitive function [216]. Recently, it has also been reported that tau is responsible for astrocyte senescence, which further contributes to synaptic dysfunction and neuronal loss in AD [217]. In addition, tau has a direct role in the induction of astrocyte senescence, probably through p53 activation [207].

Senescent astrocytes also have an impact on dopaminergic neurons, which are predominantly affected in PD. It has been shown that senescent astrocytes are able to suppress the proliferation and migration of neural precursor cells (NPCs) and reduce the viability of dopaminergic neurons [206]. In addition, the inhibition of astrocyte senescence with antioxidant Astragaloside IV prevents the degeneration of dopaminergic neurons in PD [218]. Shankar et al. proposed that the adoption of the pro-inflammatory phenotypes of senescent astrocytes might contribute to development of PD neuropathology [206]. Verma et al. suggest that α-synuclein pre-formed fibrils (α-syn PFF) induce the senescence of astrocytes, which further contributes to PD pathology [219]. Additionally, toxin 2,3,7,8-tetrachlorodibenzodioxin (TCDD), which is suspected to be a cause of PD, was shown to act through senescence astrocytes via WNT/β-catenin- and ROS-dependent mechanisms [220,221,222].

In ALS animal models, as well as in ALS patients, senescent astrocytes become toxic for motor neurons [186,223,224]. In a rodent transgenic model of ALS, the accelerated induction of astrocyte senescence phenotypes was observed, and these astrocytes provided less support to motor neurons [224]. The loss of normal astrocytic function, including the gain of the senescence phenotype, underlies cellular injury and neurodegeneration in ALS [225]. It is suggested that increased levels of SASP factors, released from senescent astrocytes, may contribute to the chronic inflammatory environment observed in ALS [226].

Although there is no confirmed role of senescent astrocytes in the development of MS, recently it has been shown that pro-inflammatory and senescence-like phenotypes of aged astrocytes have a negative impact on their supportive role in oligodendrocyte differentiation [227]. Papadopolous et al. also suggested that age-related senescent astrocytes may contribute to the progression of MS through the promotion of neuronal dysfunction and degeneration [228].

These results suggest that senescent astrocytes evidently impact NDs, such as AD, PD and ALS, and may contribute to the development of MS, but the mechanisms are still foggy, and future studies are needed to clarify the precise role of senescent astrocytes in age-related diseases of the brain.

## 4. Model Systems for the Study of Astrocyte Physiology and Pathology

The progress to be made towards the better understanding of the molecular mechanisms underlying astrocyte reactivity and senescence, and their impact on the surrounding neuronal cells, relies profoundly on adequate model systems. However, improved tools for the molecular, genetic, morphological and physiological assessments of astrocytes within the adult vertebrate CNS in vivo have been developed recently, or have been adapted from their original purposes to study neurons, as reviewed by Yu X et al. [229]. The specificity of astrocytic capturing and profiling has been provided by newly developed astrocyte-specific transgenic lines. For instance, the translating ribosome affinity purification (TRAP) approach has been used to isolate and validate the translatomes of astrocytes. This methodology enables the isolation of the messenger RNA fragments attached to the tagged ribosome, which correspond to the expression levels of various genes actively being translated. In particular, an Aldh1l1 bac-TRAP line was used to identify brain-region-dependent transcriptional differences in astrocytes across the mouse lifespan [230]. RiboTag is a related method that enables the isolation of ribosome-associated mRNAs from any cell type by an available Cre driver [231]. For instance, adult and aged astrocyte transcriptomes from different brain regions were obtained using RiboTag mice crossed with Gfap-Cre mice [232].

**Figure 6:**
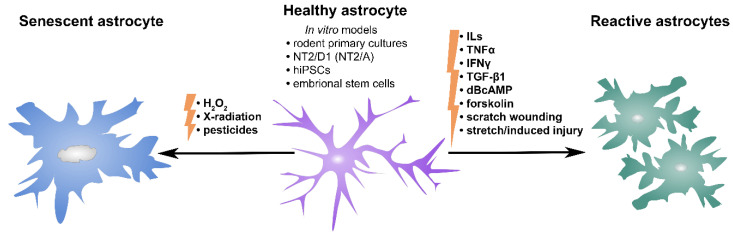
In vitro model systems for the study of astrocyte physiology and pathology. Proposed sources for in vitro models of healthy astrocytes include rodent primary cultures, astrocytes derived from human pluripotent embryonal teratocarcinoma NT2/D1 cell line (NT2/A), human-induced pluripotent stem cells (hiPSCs), and embryonal stem cells. Reactive and senescent astrocyte phenotypes can be induced by applying different stimuli on healthy astrocyte; ILs—interleukins: TNFα—tumor necrosis factor α; IFNγ—interferon γ; TGF-β1—transforming growth factor β1; dBcAMP—dibutyryl cyclic AMP; H_2_O_2_—hydrogen peroxide.

The lack of appropriate techniques for astrocyte purification and culturing has long delayed the study of their function. An important step forward occurred with the development of an astrocyte culture preparation from rodent neonatal brains [233] when it became possible to study astrocytes in a dish. Astrocyte reactivity is often induced by cytokine treatment, such as interleukins, TNFα, interferon gamma (IFNγ), or TGF-β1 [2,41,43,234], alone or in combinations [234,235]. Several in vitro injury models, including scratch wounding [236] and stretch-induced injury [237], were used to mimic different aspects of CNS trauma and to induce widespread astrocyte reactivity. Protein kinase C-activating agents, e.g., dibutyryl cyclic AMP and forskolin, were also used to induce astrocyte reactivity in vitro, documented by a reorganization of intermediate filaments [236,238,239]. Similarly, different stressors including hydrogen peroxide, X radiation, and pesticide have been used to induce astrocyte senescence (Figure 6) [177,240,241].

A key benefit of using astrocytes in culture is the absence of other cell types, providing a simplified model to study astrocyte-specific processes. This is a very important point, bearing in mind that it has been very difficult to distinguish contributions of astrocytes from those of microglia, because both cell types become reactive upon injury and both are involved in neuroinflammation [120,242,243]. However, the use of primary astrocytes in culture has certain limitations, as reviewed by Lange et al. [244]. The main limitation of traditional two-dimensional culture systems is that astrocytes in a dish show undesired baseline reactivity, even in the absence of stimuli. This occurs most due to the physical stress associated with astrocyte isolation, or due to the fundamentally different growing conditions from the in vivo niche, including the presence of serum in the culture medium [244]. Some of these problems can now be overcome with the use of modified methods for astrocyte preparation [245]. This immunopanning-based method allows the rapid isolation of pure astrocytes from the postnatal rodent brain, which are then further maintained in serum-free media. These immunopanned astrocytes retain their resting in vivo gene profiles and, unlike astrocytes obtained by initial purification and culture methods, are not highly proliferative in culture [245]. More recently, a newly developed method based on three-dimensional (3D) cell culture systems has also reduced astrocyte reactivity in vitro [246]. Indeed, astrocytes cultured on nanofiber-based 3D culture systems with bioactive coatings preserved the in vivo-like morphology and showed minimal signs of reactivity under basal conditions, but maintained full responsiveness to activation-inducing stimuli. Moreover, this bioactive 3D culture system allowed more defined cell–cell communication [246]. All of the above-mentioned advantages make this system particularly suitable for studies assessing the treatment-induced activation of astrocytes, cultured either alone or in co-cultures with other neural cells. Most of the findings on the roles of astrocytes have been obtained from rodent studies. Nevertheless, growing evidence shows that human astrocytes differ in many ways from their rodent counterparts [247,248,249]. In vitro studies with human cells have typically utilized primary astrocytes from fetal or adult tissues [249,250,251]. However, the availability of human primary astrocytes is limited, underlining the need for alternative, more accessible sources of human astrocytes for in vitro studies. Human pluripotent embryonal teratocarcinoma NT2/D1 cell line has been used as neural model system in different studies [252,253,254,255]. This is an immortalized cell line that has the ability to differentiate along the neural lineage when treated with retinoic acid, yielding both neuronal and glial cell populations [236,256]. Astrocytes derived from NT2/D1 cells (NT2/A) are similar to the primary human fetal astrocytes in their properties, including the presence of intermediate filament proteins, growth arrest, and reactive response to injury [236]. Moreover, NT2/A have an active glutamate transport system and support the growth and survival of neurons in mixed cultures [236]. These cells have been used as alternatives to human primary astrocytes in different in vitro studies [202,257]. Advancements in the field of stem cell biology have enabled the differentiation of astrocytes from human pluripotent stem cells (hPSCs) [258,259]. Moreover, the utilization of these specific protocols enabled us to selectively direct astrocyte differentiation towards a mature quiescent phenotype or a reactive inflammatory phenotype [258]. Reprogramming somatic cells into iPSCs has revolutionized the field of in vitro modelling, providing an unlimited source of human astrocytes for in vitro studies [234,260,261]. Moreover, human astrocytes obtained from the iPSCs of patients carrying a mutation of neurodegeneration-related genes have enabled better insight into the contribution of astrocytes to the pathology of underlying brain disorders, and represent an adequate model system for astrocyte-targeted drug testing [262]. Additionally, iPSCs can be integrated and differentiated within cerebral organoids to create patient-specific preclinical models. This state-of-the art technology using a 3D culture system enables the recapitulation of human-specific features of ND pathogenesis in complex tissue-like environments, including distinctive cell–cell and cell–matrix interactions required for physiological functionality [263,264]. It is expected that these human-derived models will advance our understanding of neurodegeneration from a mechanistic point of view, and also accelerate drug discovery. Indeed, cerebral organoids offer several advantages over animal models, including applicability to high throughput assays for the discovery of ND-relevant biomarkers and therapeutic targets. Moreover, cerebral organoids provide greater translatability to clinical trials, and thus accelerate the drug discovery process [263]. Organ-on-a-chip microfluidic culture devices represent the greatest advances in the field of in vitro modelling. These in vitro human microphysiological systems lined with living cells cultured under fluid flow can recapitulate organ-level physiology and pathophysiology with high fidelity [265]. Thus, newly engineered microfluidic devices have established a novel in vitro platform, enabling the controlled co-culture of neurons and astrocytes, the targeted stimulation of astrocytes, and the study of neuron–astrocyte interactions [266]. Recently, an in vitro microvascular open model system has been designed and implemented using human brain microvascular endothelial cells [267]. The greatest advantage of this human microvessel-on-a-chip platform is its compatibility with advanced imaging, including transmission electron microscopy (TEM) and the live 3D tracking of labeled molecules. This technology enables the real-time monitoring of astrocyte function, as well as real-time quantitative assessments of drug transport across brain microvessel-like barriers. Generally, this in vitro setting offers faster and simplified approaches for fundamental research and targeted drug screening that can further support the development of antibody-shuttle technologies across the highly selective human BBB [267].

All in all, advances in the field of in vitro modelling, including brain- and microvessel-on-a-chip model systems, have been emerging as powerful research tools that open new avenues for a better understanding of pathophysiology of various NDs. These cell culture models closely recapitulate crucial features of the brain environment, mimicking neuronal and glial cell interactions and integrating the effect of physiological blood flow [268], enabling a wide range of research applications, including the evaluation of disease progression, novel drug development, screening, and the non-invasive real-time monitoring of drug action [269,270,271].

## 5. Astrocytes as Promising Targets for Novel Therapeutic Approaches towards Neuroprotection and Regeneration

Since both reactive and senescent astrocytes have emerged as important players in the mechanisms underlying neurodegeneration and the progression of several NDs, they have been considered as therapeutic targets.

Astrocyte reactivity has been proposed to be a potent endogenous defense mechanism that could be manipulated to promote neuronal survival and recovery [83,101,272]. Therapeutic strategies based on the functional modulation of reactive astrocytic are aimed to enhance their beneficial roles, such as the production of neurotrophic factors, neuropoetic cytokines, and growth factors [55], while minimizing or completely eliminating undesired outcomes such as glial scar formation. For this purpose, different approaches have been applied, including viral-mediated gene therapy and the exogenous administration of compounds that induce neuroprotective functions in astrocytes [273]. For instance, it has been reported recently that the down-regulation of the JAK2/STAT pathway, specifically in the hippocampal astrocytes of transgenic APP mice, using adenoviral delivery techniques, reduces Aß deposits and improves mice spatial learning, but not memory retrieval [274]. Tools for the selective and specific delivery of therapeutics to astrocytes have been constantly refined. For instance, nanoparticles and homing peptides have been used to deliver biologically active hydrophilic compounds (e.g., proteins, peptides, nucleic acids, and small drugs) across cell and tissue barriers with high affinity and specificity for astrocytes [273]. It is interesting to mention that some drugs, normally used in clinical practice, have been shown to boost astrocyte function. A good example for this is the repurposing of US Food and Drug Administration (FDA)-approved antibiotic ceftriaxone, which has been revealed to exhibit unexpected efficiency in increasing the expression of the astrocyte-specific glutamatergic transporter EAAT2 [275]. As previously mentioned, the impairment of the glutamate transporter expression is a predisposition for excitotoxicity reported in several NDs [146,147]. Another good example is the licensed drug trifluoperazine. It has been shown that this calmodulin-inhibiting antipsychotic drug repressed AQP4 localization to the blood-spinal cord barrier, ablated AQP4-mediated cerebral edema, and led to accelerated functional recovery in rats [105]. Numerous molecules have been tested during the last years for their ability to reduce astrocyte reactivity in AD, as comprehensively reviewed by Valenza et al. [276]. However, none of them have been translated to the clinical practice yet [276].

**Figure 7:**
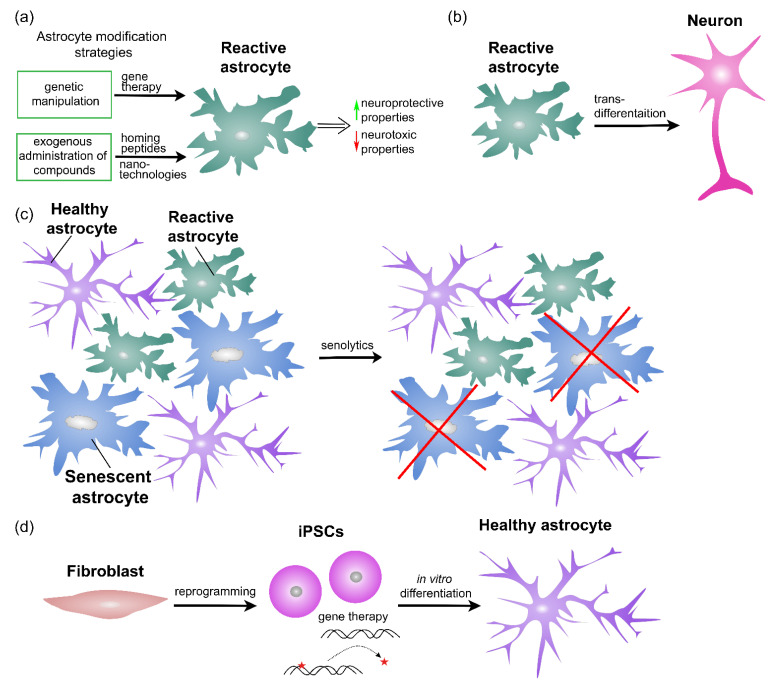
Astrocytes as promising targets for novel therapeutic approaches. (**a**) Therapeutic strategies based on the functional modulation of reactive astrocytics are aimed at enhancing their neuroprotective properties or minimizing their neurotoxic effects. For this purpose, different approaches have been applied, including viral-mediated gene therapy and the exogenous administration of compounds. Different tools including homing peptides and nanoparticles have been used for the selective and specific delivery of therapeutics to astrocytes. (**b**) Transdifferentiation or in situ reprogramming is a process that enables the conversion of endogenous astrocytes to neurons by a forced expression of single or multiple transcriptional factors, which makes astrocytes a valuable source for in situ neuronal tissue repair. (**c**) Possible treatment options in aging and aging-related diseases rely on a new class of drugs known as senolytics that work by selectively inducing apoptosis in senescent cells. (**d**) Cell replacement therapy based on the autologous transplantation of healthy astrocytes to the patients includes the following steps: the patient’s own somatic cells (fibroblasts) are reprogrammed to induce pluripotent stem cells (iPSCs); subsequently, they are genetically manipulated in vitro, then converted to healthy astrocytes and transplanted, without inducing an immune response.

The in situ reprogramming of endogenous astrocytes to neurons has appeared as a potential strategy for cellular regeneration after spinal cord injury (SCI). This is a very promising finding considering that adult spinal cord has limited ability to generate new neurons. Indeed, unlike certain regions in the adult brain where neurogenesis persists [277], the spinal cord lacks the ability to produce new neurons in adulthood [278,279]. Many studies have shown that reactive astrocytes exhibit stem cell-like properties [134,280], including the expression of neural stem cell-related proteins such as NESTIN and SOX2 [50], indicating that resident scar-forming astrocytes can be potentially transformed into neurons under specific conditions, such as the over-expression of neurogenic factors. Indeed, the conversion of reactive astrocytes to neuroblasts and neurons by the forced expression of a single transcriptional factor [281,282], a combination of multiple transcriptional factors [283], or by specific miRNAs [284] has been reported. Particularly, Su, Z et al. showed, on a mouse model with experimentally induced SCI, that ectopic SOX2 is sufficient to convert endogenous spinal astrocytes to proliferative doublecortin (DCX)-positive neuroblasts, whose survival and maturation were further enhanced by treating the mice with valproic acid [281]. Moreover, additional data have shown that, with the proper combination of neurogenic factors, it is possible to direct astrocyte transdifferentiation towards subtype-specific functional neurons (Figure 7). Thus, forced expression of the transcription factors Ngn2, Mash1 and Pax6, triggered astrocyte transition to glutamatergic neurons [285], while Dlx2 or Ascl1 induced astrocyte transdifferentiation towards GABAergic cells [286,287]. Recently, it has been demonstrated that the conversion of midbrain astrocytes into dopaminergic neurons can be performed by the antisense-induced depletion of the RNA-binding protein PTB responsible for the suppression of a neuronal induction loop. Importantly, these astrocyte-derived dopaminergic neurons reconstituted the nigral-striatal circuit, restored dopamine levels, and reversed PD-relevant motor deficits in a chemically induced mouse PD model [288]. These findings suggest that reprogrammed astrocytes have a potential to replace lost neurons and that, therefore, they represent a valuable source for in situ neuronal tissue repair. However, further research is needed to determine whether astrocyte-derived neurons can functionally replace degenerated ones.

Developing therapeutic strategies to eliminate senescent cells has also attracted attention as a possible treatment option for aging and aging-related diseases. Novel findings show that the elimination of both senescent cells and the accompanying SASP or reversal of cell senescence by genetic manipulations or pharmacological approaches may exert beneficial effects on neurodegenerative brains [210,216,289]. Notably, in vitro and in vivo approaches based on these strategies have been tested in the case of AD, as reviewed by Valenza et al. [276]. In particular, the overexpression of ∆133p53 in radiation-induced senescent astrocytes caused SASP repression [187]. Similarly, the Ginsenoside F1 in vitro treatment of D-galactose-induced senescent astrocytes suppressed SASP throughout the inhibition of p38 MAPK-dependent NF-κB activity [290]. New class of drugs known as senolytics work by selectively inducing apoptosis in senescent cells, while senomorphics are used as anti-SASP therapeutics (Figure 7) [207]. Some senolytic drugs, such as propranolol and sildenafil, hold a promise as potential therapeutics that could be beneficial for AD treatment [291,292].

An additional astrocyte-targeted therapeutic approach is based on cell replacement therapy (Figure 7). Transplanting healthy glial precursor cells, derived either from murine or human iPSCs or embryonic stem cells, has been reported to ameliorate the phenotype of rodent ALS models [293,294,295]. The establishment of iPSCs from the patient-derived somatic cells has paved the way towards personalized medicine. We could expect, in future, the application of combined gene and cell therapies to cure different NDs where the patient’s own somatic cells are genetically manipulated in vitro and then converted to healthy astrocytes and transplanted, without inducing an immune response.

## 6. Conclusions and Future Perspectives

Reactive and senescent astroglial phenotypes share many of the same features, including cellular enlargement, increased GFAP expression, the down-regulation of the glutamate transporters EAAT1 and EAAT2 and the Kir4.1 channel, and the acquisition of pro-inflammatory secretion (see Table 1). These shared features raise the possibility that many studies focused on reactive astrocytes may have actually been analyzing senescence, as already suggested by Justin Cohen and Claudio Torres [174]. However, there are accumulating data showing clear differences between these two phenotypic alternations that facilitate their distinction, as summarized in this review (Table 1, Figure 1 and Figure 4) and by others [174]. In addition, more sophisticated differences between these two phenotypes have been noted at the transcriptome and secretome levels by recent comparative analyses [241,296]. All mentioned data represent important steps towards a better understanding of the exact contribution of each of these astroglial phenotypes to brain pathology, as discussed within this review. However, there are also other challenges to be overcome, such as a clear distinction between the disease-induced dysfunction of astrocytes and their reactive response that is triggered by tissue damage. A better understanding of these diverse responses of astrocytes to injuries is important in order to elucidate the mechanisms that underlie cellular pathophysiology and govern disease progression. Additionally, it is of a great importance to understand whether the senescent phenotype itself is a consequence or a cause of different neurodegenerative processes.

Unlike neurons or oligodendrocytes, astrocyte quiescence is apparently an actively maintained state in which transcriptional and post-transcriptional mechanisms are involved in maintaining a poised state that allows rapid reactivation upon injury. Moreover, the state of astrocytes, even under non-pathological conditions, may be regulated by a balance between pro- and anti-reactive signals [104]. However, the underlying molecular mechanisms are mostly unknown, and need to be experimentally addressed. Considering that reactive astrocytes reacquire some of neural precursor properties, including the up-regulation of *Sox2* and *Sox9* expression, as well as NESTIN and VIMENTIN re-expression (Table 1), it remains to be tested whether reactive astrocytes recapture the same molecular mechanisms active during gliogenesis. Improved in vitro tools (discussed within this review) and in vivo tools (recently reviewed by [229]) for astrocyte-specific analyses and manipulation will certainly provide the answer. Given the dualistic nature of reactive astrogliosis, a comprehensive understanding of this phenomenon is of utmost importance in order to design strategies for the modulation of reactive astrogliosis to promote tissue healing and recovery. Indeed, the signaling pathways (illustrated in Figure 3) that regulate the different aspects and extent of astrogliosis remain largely unaddressed. Deeper insights into these pathways will be needed to target the specific aspects of reactive astrogliosis to maximize its beneficial effects and minimize its detrimental effects for each type of pathology. Total and single-cell multi-omics studies, including transcriptomics, proteomics, and metabolomics analyses, are very useful tools for the detection of potential therapeutic targets suitable for the efficient and precise modulation of signaling pathways. These techniques, in combination with gain-of-function and loss-of-function genetic studies, targeting specific populations of astrocytes in different humanized or animal models of specific brain pathology, will enable researchers to develop new strategies to enhance beneficial astrocytic functions and suppress the harmful ones. On the other hand, it is important to note that one of the key obstacles in the field of senescence is the lack of an appropriate biomarker that would enable accurate distinction between senescent cells and other non-dividing cells, including terminally differentiated and quiescent cells [183,297]. The identification of senescent cells with high precision and specificity would contribute to the better understanding of their impact on brain pathologies. Moreover, the existence of adequate biomarkers for senescent cells would also contribute to the improvement of targeted therapy, based on senolytics, towards their higher selectivity and specificity.

Although a lot of work remains to be completed before we will be able to successfully employ astrocyte-targeted treatment strategies, this is very dynamic research field that holds a great promise for future therapeutic approaches for ND treatments. This is of extreme importance knowing that, currently, all of the approved ND treatments are palliative and symptomatic therapies. Thus, the identification of viable therapeutic targets and new treatments for NDs is a major challenge in the field of drug discovery. Additionally, the accelerating increase in the number of affected people, causing a major healthcare burden on countries with aging populations, adds more pressure to tackle NDs. The slow progress in drug development for ND treatments could be partially attributed to inadequate preclinical models that have mostly relied on animals. Indeed, although animal models of disease may appear phenotypically similar to humans, the underlying molecular and cellular mechanisms are often distinct, and potential therapeutic targets identified in these models may, therefore, lack clinical relevance [265,298]. However, emerging advances in high-throughput screening platforms [298], coupled with breakthroughs in in vitro modeling using the advantages of iPSCs, in combination with the advancements in microfluidic systems and 3D culture models, could accelerate drug discovery and help to achieve more personalized treatments and effective precision medicine in the near future. Moreover, the widespread use of computational tools in novel drug discovery shows enormous potential in making the process less expensive and more effective [299].

## Figures and Tables

**Table 1:** Summarizing similarities and difference between reactive and senescent astrocytes.

Changes in Respect to “Quiescent/Healthy” Astroglial Phenotype	Reactive Astrocytes	References	Senescent Astrocytes	References
**Morphological** **changes**	- Cellular hypertrophy; - Changes in expression of cytoskeletal intermediate filaments;	[43]	- Enlarged, flattened, and vacuolized morphology;	[178]
	- Increased GFAP;	[40]	- Increased GFAP;	[181]
	- NESTIN and VIMENTIN up-regulation.	[41,50,51]	- Decrease in Lamin-B1 and loss of nuclear circularity/invaginated nuclei.	[179]
**Functional** **changes**	- Increased proliferation and migration;	[61]	- Permanent cell cycle arrest;	[183]
	- Secretory phenotype acquisition (the secretion of proinflammatory factors as well as neurotrophic/neuropoetic and growth factors).	[55,300]	-Senescence-associated secretory phenotype (SASP) (manly proinflammatory).	[191]
**Molecular** **changes**	- Increased expression of SOX2 and SOX9 transcription factors;	[64,66]	- Increased SA-β-gal;	[174,178,184,192]
	- Up-regulation of miR-21 and down-regulation of miR-145 expression;	[70,71]	- Increased secretion of HMGB1;	[177]
	- The down-regulation of the glutamate transporters EAAT1 and EAAT2 and the Kir4.1 channel;	[13]	- Down-regulation of EAAT1/EAAT2; Kir4.1 and AQP4;	[177]
	- Up-regulation of AQP4.	[72,73]	- The increased expression of cell cycle inhibitors p21 and p16;	[301]
			- Diminished expression of Δ133p53 and increased expression of p53β.	[186]
